# Molecularly Imprinted SERS Plasmonic Sensor for the Detection of Malachite Green

**DOI:** 10.3390/bios15050329

**Published:** 2025-05-20

**Authors:** Hao Zhang, Dani Sun, Yuhao Wen, Mengyuan Wang, Jingying Huang, Ziru Lian, Jinhua Li

**Affiliations:** 1 Marine College, Shandong University, Weihai 264209, China; zhanghao798940@163.com; 2 Coastal Zone Ecological Environment Monitoring Technology and Equipment Shandong Engineering Research Center, Shandong Key Laboratory of Coastal Environmental Processes, Laboratory of Coastal Environmental Processes and Ecological Remediation, Yantai Institute of Coastal Zone Research, Chinese Academy of Sciences, Yantai 264003, China; sun19961212@163.com (D.S.); wenyuhao115@163.com (Y.W.); wmy18963590554@163.com (M.W.); jyhuang@yic.ac.cn (J.H.); 3 University of Chinese Academy of Sciences, Beijing 100049, China

**Keywords:** malachite green, molecular imprinting, surface-enhanced Raman spectroscopy, plasmonic sensor, seawater

## Abstract

Malachite green (MG) is a highly toxic dye commonly used in industries and aquaculture, leading to significant environmental contamination and health hazards. Therefore, sensitive and selective detection of MG in real samples is urgently needed. This study presents the development of a molecularly imprinted surface-enhanced Raman spectroscopy (MI-SERS) plasmonic sensor for the rapid and sensitive detection of MG. The sensor consists of a gold nanostar (Au NS) layer as the SERS substrate and an imprinted polydopamine layer containing specific recognition sites for MG. Taking full advantage of the plasmonic effect of SERS and selective recognition capability of imprinted materials, under optimized conditions, the sensor demonstrated high sensitivity, with a detection limit of 3.5 × 10^−3^ mg/L, excellent selectivity against interference from other organic dyes, and robust performance with recoveries of 90.2–114.2% in real seawater samples. The MI-SERS sensor also exhibited good reproducibility, stability, and reusability. These findings suggest that the MI-SERS sensor is a promising tool for real-time monitoring of MG contamination in complicated samples.

## 1. Introduction

Malachite green (MG) is a widely used organic dye and veterinary drug. It can enter soils and aquatic environments through improper channels, such as industrial wastewater discharge, leaks during dye production, and aquaculture practices, leading to environmental contamination [1]. This pollution adversely affects the quality of agricultural soils and water and may even pose a threat to drinking water safety. MG persists in seawater due to its slow degradation rate, leading to prolonged contamination and alterations in the physicochemical properties of marine environments [2]. These changes contribute to the degradation of aquatic ecosystems and the destabilization of ecological balance. MG suppresses the growth of aquatic organisms, such as fish and shellfish, significantly reducing reproductive success and, in severe cases, causing mortality. Additionally, MG is highly carcinogenic and, once absorbed by aquatic organisms, can bioaccumulate through the food chain, adversely impacting higher trophic level organisms and ultimately posing significant risks to human health [3]. Therefore, effective control measures should be implemented for the production, use, and disposal of MG, along with strengthened regulatory supervision and the development of multiple detection methods.

The traditional detection methods, such as high-performance liquid chromatography (HPLC), gas chromatography–mass spectrometry (GC-MS), and ultraviolet-visible spectrophotometry (UV-Vis), are currently the most widely used in the field of MG detection [4,5,6]. These methods offer high sensitivity and accuracy, but their application often requires complex sample pretreatment and advanced instrumentation, which limits their practicality in real-time monitoring.

Recently, surface-enhanced Raman spectroscopy (SERS) has made significant advances, demonstrating its great potential and wide-ranging applications [7]. By precisely designing and controlling nanostructures, and utilizing self-assembly and nanoimprinting techniques, the SERS effect has been optimized and enhanced [8,9]. Theoretical research on the SERS surface enhancement effect has also made important progress, including the mechanisms of molecular and nanostructure interactions, electric field enhancement, and surface plasmon resonance, providing valuable insights for improving SERS substrate design [10]. To meet the growing demand for SERS technology in fields such as environmental monitoring, biomedicine, and food safety, researchers are developing new real-time, in situ, and high-throughput analysis techniques, including SERS detection systems based on microfluidic chips, laser-induced breakdown spectroscopy, and near-field Raman microscopy [11,12]. These advancements enable high-sensitivity, real-time, and in situ SERS analysis of complex biological and environmental samples.

MG contains functional groups, such as a benzene ring and ethoxyethyl groups [13]. When MG is exposed to excitation light, the vibrations of the chemical bonds within the molecule cause a temporary change in its dipole moment, leading to Raman scattering [14,15]. During this process, characteristic vibrational frequencies and intensities are generated, forming a unique Raman spectral fingerprint. This fingerprint can be used to characterize the molecular structure and chemical properties of MG [16].

However, a drawback of regular SERS is its low selectivity in complex samples, as background substances can interfere with the detection accuracy [17,18]. To solve this problem, molecularly imprinted polymers (MIPs) can be integrated with SERS; that is, molecularly imprinted SERS (MI-SERS) sensors are constructed to provide highly sensitive and selective analytical platforms [19,20]. In the recognition event by MIPs, cavities act as the “lock”, allowing the polymer to bind only to molecules that match the cavities in shape, size, and chemical functionality, just like a key fits into a lock [21,22]. Traditional MIPs still face several challenges, such as low binding efficiency, limited binding capacity, and poor accessibility of recognition sites [23]. The surface-imprinting technique (SIT) helps to solve the problem of binding sites being buried inside MIPs [24]. It uses special materials to bring the binding sites to the surface. This increases the surface area, which improves the ability to bind to the target molecule. SMIT also helps organize the binding sites better, which improves how well the polymer recognizes molecules and its overall performance [25].

To address the need for sensitive and selective MG detection in complex environmental matrices, we carefully selected gold Au NSs and polydopamine (PDA) as the core components of our MI-SERS sensor. Au NSs were chosen as the SERS substrate due to their branched morphology, which creates abundant “hotspots” at the sharp tips and junctions. These nanostructures significantly enhance the local electromagnetic field and amplify Raman signals, enabling ultra-sensitive detection. Compared with spherical or rod-shaped nanoparticles, Au NSs offer superior SERS performance due to their high tip density and anisotropic plasmonic response [26]. Additionally, gold is preferred over other metals, such as silver or copper, due to its superior stability and resistance to oxidation, which ensures that the sensor maintains high reproducibility and long-term reliability. Gold’s non-toxicity and biocompatibility further enhance its suitability for environmental and biological applications, making it an ideal material for real-world detection of trace pollutants like MG [27]. Polydopamine, inspired by the adhesion mechanism of mussel proteins, was employed as the molecular imprinting material. It can form uniform, stable, and nanoscale thin films through self-polymerization under mild alkaline conditions [28]. As a surface-imprinting material, PDA facilitates the formation of well-defined recognition sites near the surface, overcoming the limitations of traditional bulk MIPs, such as buried binding cavities and slow mass transfer. Moreover, PDA provides excellent compatibility with metal nanostructures, strong chemical stability, and low template leakage [29].

In this study, a capillary MI-SERS plasmonic sensor is developed for the detection of MG in the environmental water samples. Au NSs are selected as the SERS-enhanced substrate to amplify the Raman signal of MG and improve detection sensitivity. To prepare the MIP layer over the Au NS layer, Surface Molecular Imprinting Technique is used, in which dopamine is used as the functional monomer and crosslinking agent, and MG as the template molecule. A layer of polydopamine molecular imprinting is introduced onto the surface of a sealed-glass capillary, which can accurately recognize MG molecules and improve the material’s selectivity, reducing interference from impurities. In the detection scheme, the Raman signal of the MI-SERS sensor increases with the rising MG concentration, enabling quantitative detection of MG.

## 2. Materials and Methods

### 2.1. Reagents and Materials

MG, crystal violet, and rhodamine B were purchased from China National Pharmaceutical Group Chemical Reagents Co., Ltd. (Shanghai, China). N-2-hydroxyethylpiperazine-N′-2-ethanesulfonic acid (HEPES) and dopamine hydrochloride powder were purchased from Beijing Solarbio Science & Technology Co., Ltd. (Beijing, China). Tetrachloroauric acid (HAuCl_4_·_4_H_2_O), (3-aminopropyl)triethoxysilane (APTES), sulfuric acid (H_2_SO_4_, 98.08%), hydrogen peroxide (H_2_O_2_, 30%), acetic acid (CH_3_COOH, 99.5%), anhydrous ethanol (99.7%), sodium dodecyl sulfate (SDS), and tris(hydroxymethyl)aminomethane hydrochloride (Tris-HCl) were obtained from Chengdu Xiya Chemical Technology Co., Ltd. (Chengdu, China) The ultrapure water used in the experiments was prepared with a Millipore water purification system (Burlington, MA, USA), with a resistivity of 18.2 MΩ·cm, and was used immediately. Glass capillaries with an inner diameter of 0.5 mm and a length of 100 mm were provided by West China Medical University Instrument Factory (Chengdu, China).

### 2.2. Apparatus

The microscope confocal Raman spectrometer (Renishaw inVia, UK) was purchased from Renishaw (Shanghai, China) Trading Co., Ltd., and the pH-3c digital pH meter was purchased from Shanghai Leici Instruments Factory. The DZF-6050 vacuum drying oven was purchased from Shanghai Boxun Industrial Co., Ltd. (Beijing, China). The HH-420 electric constant-temperature water bath was purchased from Shanghai Jinghong Experimental Equipment Co., Ltd. (Shanghai, China). The TG16-WS centrifuge was purchased from Hunan Xiangyi Laboratory Instrument Development Co., Ltd. (Changsha, China) The scanning electron microscope (SEM) was from Hitachi S-4800. (Tokyo, Japan)

### 2.3. Construction of the MI-SERS Sensor

#### 2.3.1. Functionalization of Glass Capillaries

A blowtorch flame was used to uniformly seal both ends of the glass capillary. The capillary was placed into a test tube containing ultrapure water for 30 min, and its airtightness was checked to ensure that subsequent treatments occurred entirely on the surface of the glass capillary. The absence of a water column at both ends indicated good airtightness. The test tube was then placed into a dry test tube, 7 mL of concentrated sulfuric acid was added to the test tube, followed by 3 mL of hydrogen peroxide, and the mixture was well mixed. The glass capillary was submerged in the H_2_SO_4_-H_2_O_2_ (7:3, *v*/*v*) solution and left to stand at room temperature for 4 h in a fume hood. After repeatedly rinsing with ultrapure water, the moisture content was controlled, and the test tube with the glass capillary was placed in a 75 °C drying oven to dry overnight, completing the hydroxylation process.

Then, the glass capillary underwent amino functionalization. We added 1 mL of APTES to the test tube, followed by 24 mL of ethanol, and immersed the cleaned glass capillary vertically into the APTES-ethanol solution. We sealed the test tube and heated it in a 70 °C oil bath for 6 h to complete the silanization process. Then, we washed the glasses with ethanol to submerge the capillary, sonicated for 15 min, and repeated 3 times to remove any unreacted silane. Finally, we dried the capillary in a vacuum drying oven at 100 °C for 2 h, then removed and stored it sealed at room temperature.

#### 2.3.2. Preparation of the SERS Substrate

A 0.1 mol L^−1^ HEPES standard aqueous solution was prepared using ultrapure water. The pH was adjusted to 7.5 ± 0.5 at room temperature by adding a 1 mol L^−1^ NaOH solution. Au NSs were synthesized via the one-pot method. First, 2 mL of 0.1 mol L^−1^ HEPES (pH 7.5) solution was mixed with 3 mL of ultrapure water, followed by the addition of 40 μL of 24.25 mmol L^−1^ HAuCl_4_ solution. The mixture was shaken at room temperature and then immediately placed in a 25 °C water bath for 20 min. During this time, the solution changed color from pale yellow to colorless, pink, purple, and finally deep blue, indicating successful synthesis [30].

The glass capillaries were immersed in the above Au NSs colloidal suspension for 9 h, forming an Au NSs layer on the outer surface of the glass capillary (visible to the naked eye). The glass capillary was then gently washed with ultrapure water, completing the synthesis of the SERS-active glass capillary.

#### 2.3.3. Preparation of the Polydopamine-Imprinted Layer

**Figure 1:**
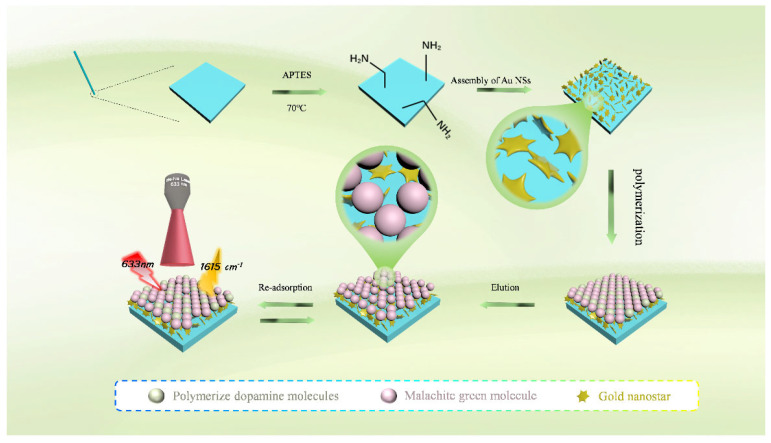
Schematic illustration of MI-SERS sensor construction.

The entire material preparation process is shown in Figure 1. We dissolved 1.5 mg of MG and 4 mg of hydrochloric acid dopamine in 20 mM Tris-HCl buffer (pH 8.5). Then, we immersed the glass capillary coated with the SERS substrate vertically into the above solution and let it stand in a 25 °C constant-temperature water bath. Raman signals were detected every 10 min. When the signal increased and no longer changed, it indicated that the target molecule had completely occupied the cavity, and the polymerization reaction was complete (40 min). After polymerization, the capillary sensor was washed by soaking it in an elution solution (ethanol:ammonia:water = 7:2:1) for 5 min. This process was repeated twice, removing the template molecules from the polydopamine-imprinted layer. Then, we soaked the capillary sensor in ultrapure water for 5 min and changed the water twice to thoroughly wash away any remaining ethanol and ammonia. The control group, the nonimprinted SERS sensor (NI-SERS), was constructed in the same way as the MI-SERS sensor but without the addition of the template molecule.

The quantitative detection of MG consisted of the following two steps: First, we immersed the tip of the sensor into 200 µL of sample solution containing MG and incubated it for 20 min for target molecule recognition. Next, we used a microconfocal Raman microscope to perform SERS detection on the glass capillary sensor. The laser wavelength was 633 nm, the laser power was 20 mW, the exposure time was 1 s, and the accumulation time was 2 times.

### 2.4. Performance Testing of the MI-SERS Sensor

In environmental wastewater, multiple organic dyes may be present simultaneously [31]. To evaluate the specificity of the Raman peak at 1615 cm^−1^ for detecting MG, crystal violet and rhodamine B, which have stronger Raman signals than MG, were chosen as interfering substances. The MI-SERS sensor was tested under the same conditions for detecting these interfering substances, as well as for detecting the three substances simultaneously. In the reproducibility test, thirty points were randomly selected from five different batches of sensors to measure the SERS intensity. Additionally, the MI-SERS sensor’s signal variation was tested under long-term storage conditions (18 days) both in a vacuum and under ambient conditions. All SERS spectra were baseline corrected, and Prism 8 software (GraphPad, USA) was used for graph plotting and spectral linear analysis. The actual samples were taken from the Yantai University seaside bathing area, filtered, and then directly spiked for the actual sample testing.

## 3. Results and Discussion

### 3.1. Construction and Condition Optimization of MI-SERS Sensor

The construction steps of the MI-SERS sensor were as follows: Charged gold nanoparticles were electrostatically assembled on the surface of an amino-functionalized glass capillary to form a SERS-active substrate, which was used to enhance the Raman signal of malachite green. Subsequently, a polydopamine (PDA) layer with controllable thickness was uniformly applied onto the gold nanoparticles via biomimetic imprinting, and the PDA layer contained malachite green molecules. After the removal of the malachite green molecules, imprinting of the PDA created selective binding cavities, which were responsible for protein recognition. Once the MI-SERS sensor was constructed, it could directly be used for the detection of malachite green molecules by exploiting the Raman shift of the malachite green molecules themselves.

**Figure 2:**
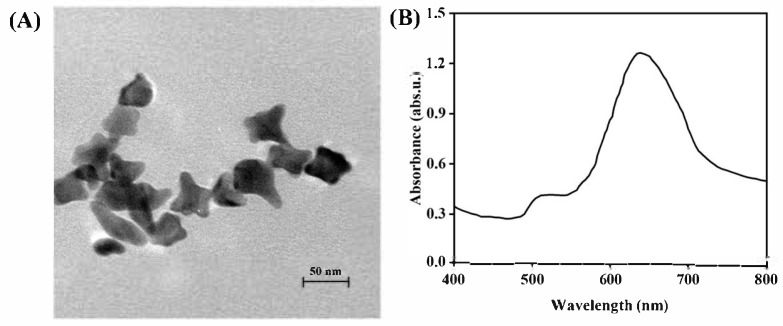
(**A**) TEM image of Au NSs. (**B**) UV-Vis spectrum of Au NSs.

Au NSs were prepared using a one-pot method to achieve high-performance substrates for SERS. At the tips of the Au NSs, polarity enhancement can occur, creating “hotspots” that generate strong SERS signals. From the TEM images (Figure 2A), it can be seen that Au NSs were a mixture of 3D nanoparticles of various shapes, including spheres and branched particles with different numbers of tips [26]. These structures provided numerous hotspots that were essential for generating intense SERS signals. The Au NS solution exhibited two surface plasmon resonance (SPR) peaks at 510 and 638 nm (Figure 2B). The former was likely attributed to the transverse plasmon resonance of the nanostar tips, while the latter originated from the longitudinal plasmon resonance of the elongated branches of the nanocrystals.

**Figure 3:**
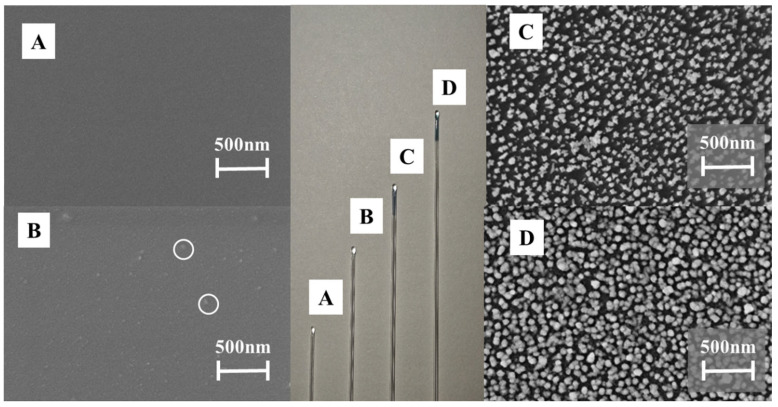
SEM images and photo-images (middle) of the glass capillary, (**A**) Glass capillary treated with piranha solution; (**B**) Glass capillary with introduced hydroxyl groups; (**C**) Glass capillary with introduced Au NSs; (**D**) MI-SERS sensor.

We observed the following through the characterization of the MI-SERS sensor: The glass capillary tube had a smooth surface (Figure 3A). After modification, the surface showed regularly distributed, particle-like protrusions (Figure 3B). After self-assembly of the glass capillary tube with Au NSs, the SERS-active substrate was evenly coated on the capillary’s surface (Figure 3C). The PDA imprinting layer was uniformly coated on the Au NSs’ active substrate surface (Figure 3D).

To achieve the optimal performance of the MI-SERS sensor, a systematic optimization was conducted on the effects of various experimental parameters on the sensor’s performance. Several experimental variables that significantly affected the sensor’s performance were optimized. Key parameters, such as the mass ratio of the target molecule to dopamine, the composition and duration of the elution solution, and the polymerization time, were investigated. The performance of the MI-SERS sensor was evaluated using the intensity of the 1615 cm^−1^ peak as the analytical signal, with five parallel experiments conducted.

**Figure 4:**
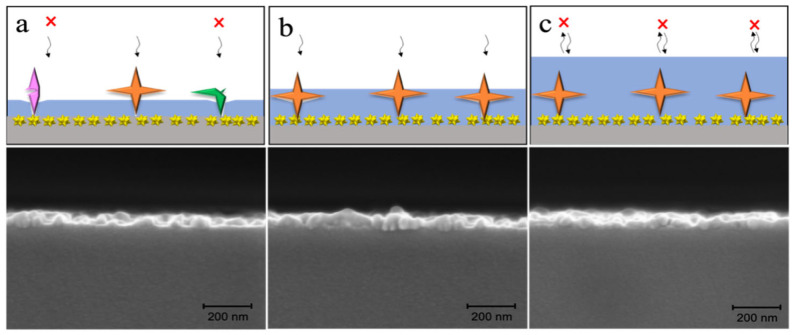
(Top) Schematic diagram of the effect of PDA imprinting layer thickness on target identification and mass transfer at different ratios of MG to PDA of (**a**) 15/25, (**b**) 15/30, and (**c**) 15/40. (Bottom) The corresponding cross-section SEM images of the polymerized dopamine coating.

By adjusting the amount of the target molecule MG and PDA in a specific ratio, the thickness of the polymerized PDA imprint layer on the substrate surface could be further controlled, thus obtaining an imprint layer that included the necessary binding sites. Incomplete crosslinking of the PDA would make the cavities prone to deformation, reducing selectivity (Figure 4a). Therefore, it was essential to ensure the retention of the imprint cavity after template removal (Figure 4b). Meanwhile, excessive crosslinking of PDA would hinder the elution and rebinding of MG (Figure 4c). Experiments were conducted with ratios of 15/25 mg, 15/30 mg, 15/40 mg, and 15/45 mg, resulting in an imprint layer of gradually increasing thickness on the surface of Au NSs. The results showed that the best linear relationship was obtained when the MG/PDA ratio was 15/40 mg. Cross-sectional SEM images confirmed that the polymerized PDA imprint layer was successfully coated on the surface of Au NSs, with 15/40 mg being the optimal mass ratio.

**Figure 5:**
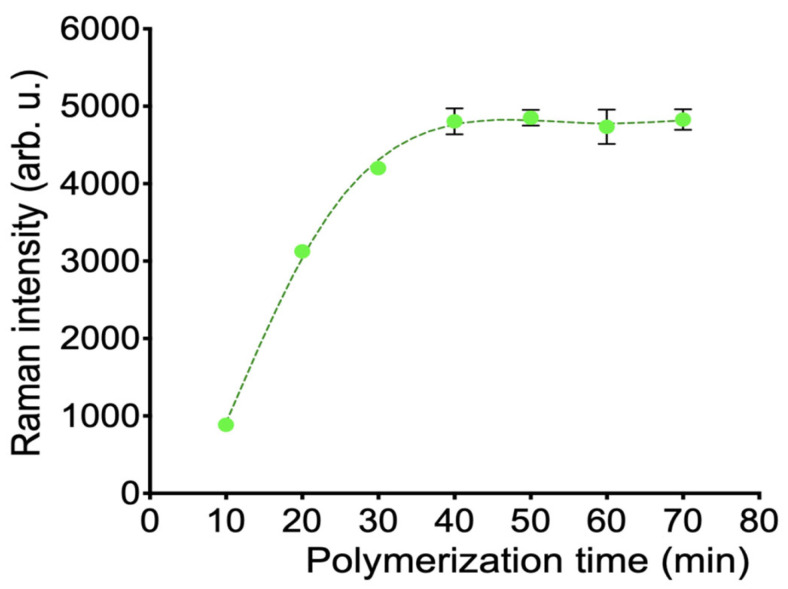
Raman signal intensity changes with polymerization time.

In an alkaline environment, PDA readily undergoes oxidative polymerization. The solution oxidation polymerization method is simple, does not require complex instruments, and was chosen to construct the MI-SERS sensor. By simply controlling the polymerization time, the thickness of the PDA film could be adjusted at the nanoscale. The chemical composition of the buffer solution could effectively regulate the polymerization of PDA. The optimal polymerization time was determined by the Raman signal peak. As the Raman peak at 1615 cm^−1^ showed the strongest intensity for MG, we selected this specific peak for the time optimization analysis. As shown in Figure 5, with the increase in polymerization time, the Raman signal gradually strengthened. At 40 min, the signal no longer changed, indicating the optimal polymerization effect had been reached.

After imprinting, it was necessary to remove the template molecules to obtain specific cavities. Therefore, the elution strength of the solvent must both ensure the stability of the physical and chemical properties of the polydopamine-imprinted layer and completely remove the template molecules. In this regard, different elution solvents were tested, including combinations and ratios of methanol, ethanol, acetic acid, ammonia solution, etc. The results showed that a mixed solution of ethanol, ammonia solution, and water (7:2:1) could completely remove the template molecules. Additionally, the number of cyclic elution steps in the range of 1 to 5 was studied, and two repetitions of elution were chosen as the optimal condition. Subsequent experimental results confirmed that the elution solution did not affect the rebinding of the target molecule to the imprint cavity, and no over-elution phenomenon occurred, ensuring no impact on the stability of the imprint layer.

### 3.2. Selectivity and Anti-Interference Ability of the MI-SERS Sensor

**Figure 6:**
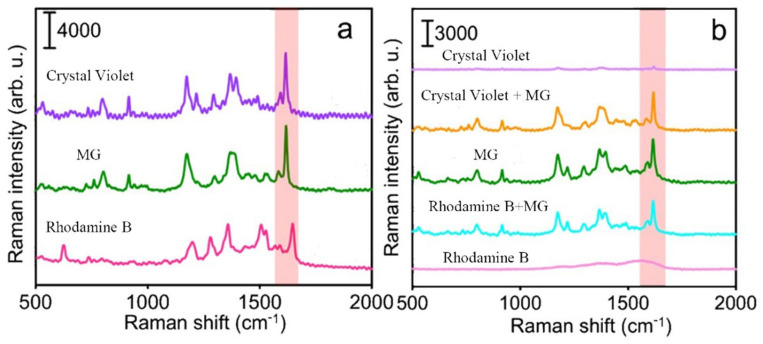
(**a**) SERS spectra of MG, crystal violet, and rhodamine B, respectively, and (**b**) comparison diagram of the MI-SERS sensor in detecting MG, crystal violet, rhodamine B, and their mixed solutions.

As shown in Figure 6, the characteristic shifts of MG occurred at 795, 1215, and 1615 cm^−1^, and the Raman peak at 1615 cm^−1^ was the strongest. Therefore, this Raman peak at 1615 cm^−1^ was selected as the characteristic peak for analysis, as it provided the highest sensitivity for MG detection. In this experiment, the concentrations of MG, crystal violet, and rhodamine B were all 5 ppm, and SERS detection was performed using the MI-SERS sensor under the same conditions. The SERS intensities near the 1615 cm^−1^ characteristic peak for different analytes were compared. As shown in Figure 6a, crystal violet and rhodamine B both had characteristic peaks near this region, which could potentially interfere with the detection of MG. Therefore, the MI-SERS sensor was incubated in a mixed solution of MG with crystal violet and rhodamine B for detection. The results were compared to the signals obtained from the individual detections of MG, crystal violet, and rhodamine B solutions. The results showed that under repeated testing with all three samples, crystal violet and rhodamine B had no significant effect on the SERS response of MG. The MI-SERS sensor, using MG as the template molecule, also did not produce any signal from these two organic dyes, indicating that the MI-SERS sensor has strong anti-interference ability and selectivity (Figure 6b).

### 3.3. Sensitivity of the MI-SERS Sensor

**Figure 7:**
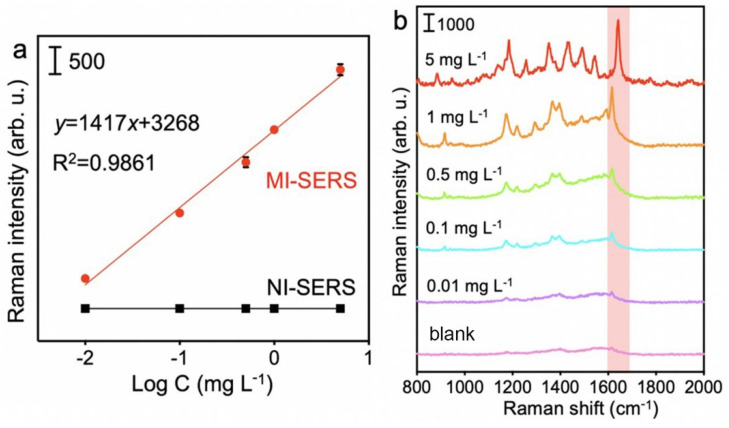
(**a**) The linear relationship between the Raman intensity of MI-SERS and NI-SERS sensors and the logarithm of MG concentration. The error bar represents the standard deviation of three measurements. (**b**) SERS spectra of MG on the MI-SERS sensor at different concentrations.

By optimizing the main factors affecting the sensor’s performance, we further investigated its sensitivity. Under optimal conditions, at the 1615 cm^−1^ characteristic peak, the Raman signal intensity showed a good linear relationship with the logarithm of the MG concentration in the range of 0.01 mg/L to 5 mg/L (*y* = 1417*x* + 3268), with a correlation coefficient (R^2^) of 0.9861 (Figure 7a).

Figure 7b shows the SERS spectra of different concentrations of MG detected by the MI-SERS sensor. The Raman signal intensity significantly decreased as the MG concentration decreased. The limit of detection (LOD) was 3.5 × 10^−3^ mg/L, and the calculation process was as follows: LOD = 3*σ*/s, where *σ* represents the standard deviation of 11 measurements of the blank group, and *s* refers to the slope of the standard curve. In the above standard curve, *s* was 1417.

Compared to methods such as UV-Vis and HPLC, the LOD was almost in the same order of magnitude [32]. However, compared to other MI-SERS methods [32,33], there is still room for further improvement, particularly in terms of selectivity and stability. All in all, it is sufficient for preliminary rapid detection applications [33].

### 3.4. Reproducibility and Stability of the MI-SERS Sensor

**Figure 8:**
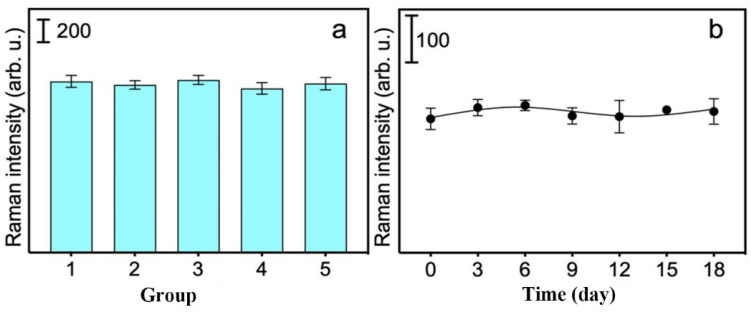
(**a**) Changes in SERS intensity between five MI-SERS sensors. (**b**) Raman signal variation over time of the MI-SERS sensor in a vacuum.

To evaluate the reproducibility of the MI-SERS sensor, five parallel sensors from five different batches were exposed to the same concentration of MG solution (0.1 ppm) under identical conditions. Thirty points were randomly selected to measure the SERS intensity. The results shown in Figure 8a indicate that the RSD of the thirty spectral data points at the characteristic peak was approximately 4.54%, demonstrating excellent reproducibility of the MI-SERS sensor.

The stability of the MI-SERS sensor was also studied under long-term storage (18 days) both in a vacuum and under ambient conditions. The signal of the MI-SERS sensor stored under ambient conditions decreased over time, indicating poor stability. We measured the SERS signal of the MI-SERS sensor stored under a vacuum at the same time intervals (every three days), and the results are shown in Figure 8b. The stability of the Au NSs may be attributed to the use of HEPES buffer as a surface stabilizer, and the vacuum environment prevented the Au NSs from changing due to exposure to air. After 18 days, the signal remained almost unchanged, and the vacuum storage environment slowed down the oxidation process of the material, thereby prolonging the sensor’s lifespan.

### 3.5. The Reusability of the MI-SERS Sensor

**Figure 9:**
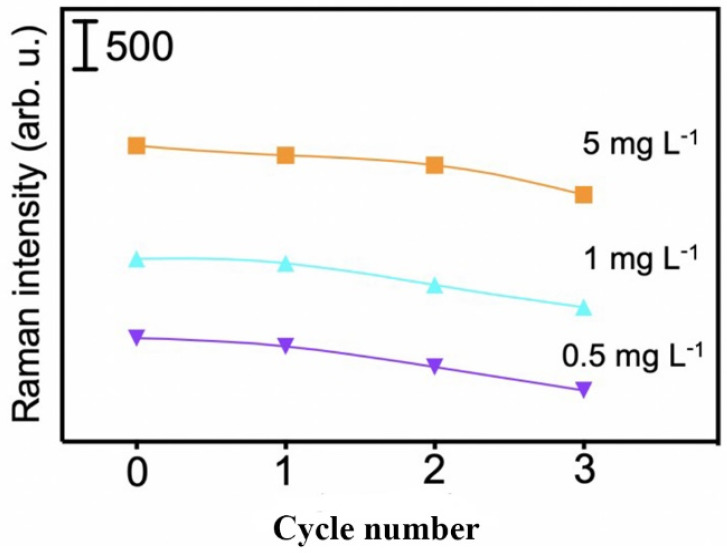
SERS intensity after three repeated cycles of elution and detection at three different concentrations.

The Au NS substrate and the PDA-imprinted layer in the MI-SERS sensor are both extremely thin, making the elution process inevitably prone to causing some degree of damage. However, the reusability of the sensor is a critical factor in evaluating its performance and recyclability. To assess this, we performed elution–repolymerization cycles at three different concentrations. After three cycles, the SERS intensity at 1615 cm^−1^ remained within 79–89% of the initial intensity (Figure 9). This result indicated that some damage to the imprinted cavities or loss of the Au NS substrate may have occurred during the process, but the variation in the SERS signal for MG was kept within an acceptable range. These findings demonstrated that the MI-SERS sensor exhibited good reusability and has the potential for repeated cycles of use.

### 3.6. Application of the MI-SERS Sensor to Actual Seawater Samples

**Figure 10:**
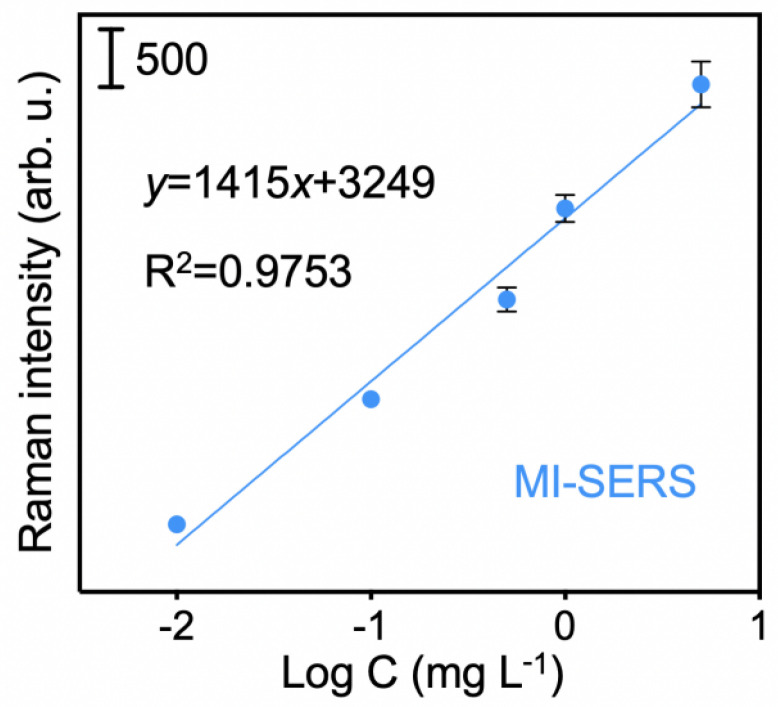
Linear relationship between SERS intensity and logarithm of MG concentration in the seawater sample.

MG was not detected in any of the three actual samples. Therefore, these samples were used as blank matrices for spiked experiments. A linear relationship between the SERS intensity and the logarithm of the MG concentration was plotted (Figure 10), which closely matched the standard curve. This indicated that the components and impurities in the seawater matrix had minimal effect on the sensor. The linear equation in the water sample, with an R^2^ of 0.9753, showed a clear linear correlation, which was similar to the standard solution calibration curve. It can be used for quantitative analysis based on the standard solution calibration curve, making it suitable for batch detection of different matrix samples and meeting the requirements for rapid testing.

Furthermore, the recovery rates were investigated, and for the three spiked concentrations, the values ranged from 90.2% to 114.2%, with relative standard deviations between 11.1% and 15.7% (Table 1). These results clearly demonstrated that the sensor is capable of rapid and accurate quantification of MG in actual seawater samples, providing a feasible method for trace detection of MG in complex matrices, such as coastal waters.

## 4. Conclusions

In this study, we developed a novel MI-SERS sensor for the rapid and sensitive detection of MG in complex environmental water samples. The sensor was constructed based on the SIT by imprinting a polydopamine layer on Au NSs. The sensor demonstrated excellent selectivity, sensitivity, and reproducibility, with a low LOD. It showed strong anti-interference ability, good stability under different storage conditions, and reusability after multiple cycles. The sensor was successfully applied to seawater samples, achieving high recovery rates and accuracy. This MI-SERS sensor offers a promising approach for real-time, in situ environmental monitoring of MG and could be further optimized for broader applications.

## Figures and Tables

**Table 1:** Determination of MG in seawater samples by the MI-SERS sensor (n = 3).

Sampling Site	Spike (µg L^−1^)	Detection (µg L^−1^) ± SD	Recovery Rate (%)	RSD
Yantai University Beach	5	5.57 ± 0.67	111.4	12.03%
	1	1.14 ± 0.18	114.2	15.79%
	0.1	0.09 ± 0.01	90.2	11.11%

## Data Availability

Detailed data can be obtained from the authors.
